# Supplementary material for: Social jetlag and sleep debts are altered in different rosters of night shift work
Source: PLoS One. 2022 Jan 7;17(1):e0262049. doi: 10.1371/journal.pone.0262049 (PMC8740972; doi:10.1371/journal.pone.0262049)

**S1 File. Representation of shift schedules of the different rosters.** M=morning shift; E=evening shift; N=night shift; D=day shift.


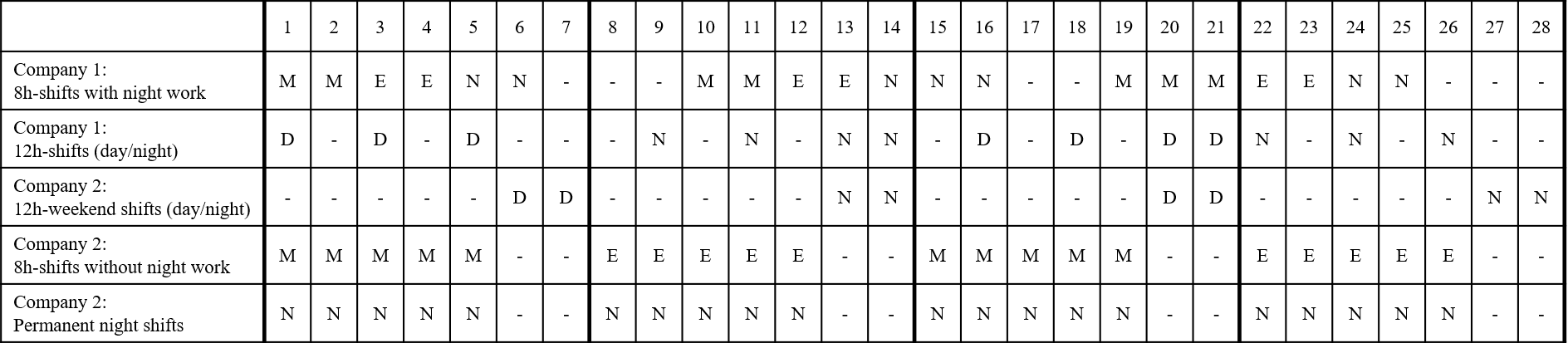

Supplement: S1 File — M = morning shift; E = evening shift; N = night shift; D = day shift. (DOCX) [file pone.0262049.s001.docx]
