# Supplementary material for: Social jetlag and sleep debts are altered in different rosters of night shift work
Source: PLoS One. 2022 Jan 7;17(1):e0262049. doi: 10.1371/journal.pone.0262049 (PMC8740972; doi:10.1371/journal.pone.0262049)
Supplement: S2 File — (DOCX) [file pone.0262049.s002.docx]

**S2 File. Additional linear mixed model analysis for LIDS levels and range of LIDS oscillation**

**Table A.** **Linear mixed model analysis for LIDS levels and range of LIDS oscillation in 117 male shift workers**

|  | LIDS level | | | LIDS oscillation | | |
| --- | --- | --- | --- | --- | --- | --- |
| Fixed effects | β | 95% CI | | β | 95% CI | |
| Intercept | 69.81 | 58.02 | 81.58 | 76.80 | 66.53 | 87.05 |
| Age (10 years) | -1.32 | -3.20 | 0.56 | -1.25 | -2.63 | 0.13 |
| Sleep duration (h) | **-0.84** | **-1.13** | **-0.54** | 0.22 | -0.41 | 0.86 |
| LIDS cycle number | **-12.98** | **-16.57** | **-9.39** | **-6.77** | **-10.76** | **-2.76** |
| Night sleep^a^ | **-3.35** | **-4.40** | **-2.28** | 1.93 | -0.37 | 4.19 |
| Work day^b^ | **-1.56** | **-2.70** | **-0.42** | 2.23 | -0.37 | 4.81 |
| Shift system^c^ |  |  |  |  |  |  |
| 12h-shifts (day/night) | **-9.05** | **-16.96** | **-1.14** | **-6.09** | **-11.88** | **-0.28** |
| 8h-shifts with night work | **-9.97** | **-17.89** | **-2.04** | **-8.45** | **-14.27** | **-2.61** |
| 8h-shifts without night work | -3.56 | -13.09 | 5.97 | -6.50 | -13.58 | 0.60 |
| Permanent night shifts | **-15.70** | **-25.08** | **-6.31** | -6.56 | -13.59 | 0.47 |
| Interaction effects |  |  |  |  |  |  |
| LIDS cycle*age | 0.34 | -0.22 | 0.90 | -0.01 | -0.49 | 0.48 |
| LIDS cycle*sleep duration | **0.66** | **0.54** | **0.77** | **0.40** | **0.12** | **0.67** |
| LIDS cycle*night sleep^a^ | **1.58** | **1.19** | **1.96** | -0.28 | -1.21 | 0.68 |
| LIDS cycle*work day^b^ | **1.06** | **0.63** | **1.48** | -0.70 | -1.81 | 0.42 |
| LIDS cycle*shift system^c^ |  |  |  |  |  |  |
| 12h-shifts (day/night) | 2.25 | -0.11 | 4.61 | 0.35 | -1.71 | 2.40 |
| 8h-shifts with night work | 2.35 | -0.02 | 4.72 | 0.94 | -1.13 | 3.02 |
| 8h-shifts without night work | -0.07 | -2.92 | 2.78 | 1.03 | -1.53 | 3.58 |
| Permanent night shifts | **4.32** | **1.51** | **7.13** | 0.69 | -1.87 | 3.26 |

CI=95% confidence interval; ^a^reference: no sleep between 8pm and 8am, ^b^reference: unrestricted sleep episodes, ^c^reference: 12h-weekend shifts (day/night)

**Table B. Comparison of sleep quality between shift workers with normal (8h-shifts with night work) and extended shift duration (12h-shifts) in company 1 assessed with linear mixed model analysis**

|  | LIDS level | | | LIDS oscillation | | |
| --- | --- | --- | --- | --- | --- | --- |
| Fixed effects | β | 95% CI | | β | 95% CI | |
| Intercept | 72.76 | 59.07 | 86.42 | 68.18 | 55.82 | 80.54 |
| Age (10 years) | -1.76 | -3.88 | 0.36 | -1.40 | -2.98 | 0.19 |
| Men^a^ | -9.89 | -21.12 | 1.35 | -2.63 | -11.35 | 6.07 |
| Sleep duration (h) | **-0.92** | **-1.25** | **-0.58** | 0.58 | -0.14 | 1.31 |
| LIDS cycle number | **-13.57** | **-17.57** | **-9.57** | **-5.60** | **-10.07** | **-1.12** |
| Night sleep^b^ | **-6.08** | **-10.01** | **-2.13** | -1.37 | -6.75 | 3.95 |
| Work day^c^ | **-1.87** | **-3.17** | **-0.56** | **4.04** | **1.04** | **7.01** |
| 12h-shifts (day/night)^d^ | 0.37 | -4.12 | 4.86 | 1.98 | -1.41 | 5.37 |
| Interaction effects |  |  |  |  |  |  |
| Male*Night sleep | 2.71 | -1.27 | 6.69 | 3.99 | -0.99 | 8.96 |
| LIDS cycle*Age | **0.62** | **0.01** | **1.24** | 0.07 | -0.45 | 0.58 |
| LIDS cycle*Male^a^ | 1.45 | -1.72 | 4.63 | -0.06 | -2.72 | 2.60 |
| LIDS cycle*Sleep duration | 0.69 | 0.56 | 0.81 | **0.34** | **0.02** | **0.66** |
| LIDS cycle*Night sleep^b^ | **1.41** | **1.00** | **1.82** | -0.26 | -1.28 | 0.77 |
| LIDS cycle*Work day^c^ | **0.98** | **0.49** | **1.46** | -1.16 | -2.44 | 0.12 |
| LIDS cycle*12h-shifts^d^ | 0.08 | -1.22 | 1.39 | -0.52 | -1.65 | 0.62 |

CI=95% confidence interval; ^a^reference: women, ^b^reference: no sleep between 8pm and 8am, ^c^reference: unrestricted sleep episodes, ^d^reference: 8h-shifts with night work

**Table C. Impact of chronotype on LIDS levels and range of LIDS oscillation assessed with linear mixed model analysis**

|  | LIDS level | | | LIDS oscillation | | |
| --- | --- | --- | --- | --- | --- | --- |
| Fixed effects | β | 95% CI | | β | 95% CI | |
| Intercept | 47.61 | 45.01 | 50.22 | 67.11 | 65.15 | 69.05 |
| Early chronotype^a^ | -2.59 | -7.30 | 2.13 | -1.32 | -4.78 | 2.14 |
| Late chronotype^a^ | -1.79 | -6.44 | 2.86 | -0.62 | -3.96 | 2.74 |
| LIDS cycle number | **-2.87** | **-3.65** | **-2.09** | **-2.76** | **-3.43** | **-2.10** |
| Interaction effects |  |  |  |  |  |  |
| LIDS cycle number*Early chronotype^a^ | -0.04 | -1.46 | 1.38 | -0.14 | -1.35 | 1.06 |
| LIDS cycle number*Late chronotype^a^ | -0.15 | -1.54 | 1.25 | 0.04 | -1.11 | 1.19 |

CI=95% confidence interval; ^a^reference: intermediate chronotype

**Table D. Linear mixed model analysis for LIDS levels stratified by chronotype**

|  | Early chronotype | | | Intermediate chronotype | | | Late chronotype | | |
| --- | --- | --- | --- | --- | --- | --- | --- | --- | --- |
| Fixed effects | β | 95% CI | | β | 95% CI | | β | 95% CI | |
| Intercept | 76.85 | 51.35 | 102.14 | 84.08 | 64.33 | 103.79 | 91.57 | 67.81 | 115.16 |
| Age (10 years) | -1.43 | -4.67 | 1.79 | -0.89 | -3.69 | 1.91 | **-5.80** | **-10.19** | **-1.41** |
| Men^a^ | 0.27 | -17.32 | 17.84 | **-12.03** | **-23.83** | **-0.23** | -4.36 | -14.36 | 5.64 |
| Sleep duration (h) | -0.33 | -0.91 | 0.30 | **-0.89** | **-1.30** | **-0.47** | **-1.01** | **-1.58** | **-0.44** |
| LIDS cycle number | **-12.95** | **-19.11** | **-6.71** | **-14.69** | **-20.96** | **-8.40** | **-13.44** | **-21.66** | **-5.13** |
| Night sleep^b^ | **-13.17** | **-20.18** | **-6.13** | **-7.74** | **-11.77** | **-3.72** | -1.60 | -6.05 | 2.95 |
| Work day^c^ | 0.40 | -1.84 | 2.69 | **-2.50** | **-4.11** | **-0.87** | -0.27 | -2.38 | 1.84 |
| Roster^d^ |  |  |  |  |  |  |  |  |  |
| 12h-shifts (day/night) | -17.17 | -41.79 | 7.46 | **-12.69** | **-24.13** | **-1.23** | -10.21 | -20.86 | 0.56 |
| 8h-shifts with night work | **-26.61** | **-51.04** | **-2.12** | -9.99 | -21.53 | 1.56 | -9.30 | -20.44 | 1.90 |
| 8h-shifts without night work | -18.20 | -41.37 | 4.95 | - | - | - | -14.22 | -35.33 | 6.96 |
| Permanent night shifts | **-30.27** | **-59.28** | **-1.26** | -13.72 | -29.07 | 1.63 | **-11.20** | **-20.79** | **-1.55** |
| Interaction effects |  |  |  |  |  |  |  |  |  |
| Male*Night sleep | **11.43** | **4.30** | **18.64** | 2.28 | -1.77 | 6.34 | 2.69 | -2.03 | 7.32 |
| LIDS cycle number*age | 0.61 | -0.16 | 1.39 | 0.39 | -0.50 | 1.27 | 1.07 | -0.47 | 2.60 |
| LIDS cycle number*men^a^ | **-6.36** | **-10.52** | **-2.21** | 1.27 | -2.43 | 4.98 | -1.13 | -4.55 | 2.29 |
| LIDS cycle number*sleep duration | **0.49** | **0.24** | **0.73** | **0.75** | **0.59** | **0.91** | **0.58** | **0.37** | **0.78** |
| LIDS cycle number*night sleep^b^ | ***0.69*** | -0.07 | 1.45 | **2.21** | **1.70** | **2.72** | -0.16 | -0.96 | 0.63 |
| LIDS cycle number*work day^c^ | 0.47 | -0.36 | 1.30 | **1.70** | **1.09** | **2.31** | -0.04 | -0.83 | 0.75 |
| LIDS cycle number*roster^d^ |  |  |  |  |  |  |  |  |  |
| 12h-shifts (day/night) | **9.30** | **3.44** | **15.16** | 1.51 | -2.10 | 5.12 | 1.40 | -2.40 | 5.12 |
| 8h-shifts with night work | **8.89** | **3.06** | **14.69** | 1.38 | -2.27 | 5.02 | 2.02 | -1.89 | 5.90 |
| 8h-shifts without night work | **7.27** | **1.79** | **12.77** | - | - | - | 3.44 | -3.95 | 10.79 |
| Permanent night shifts | **11.49** | **4.51** | **18.48** | 1.88 | -2.98 | 6.74 | **3.74** | **0.35** | **7.10** |

CI=95% confidence interval; ^a^reference: women; ^b^reference: no sleep between 8pm and 8am; ^c^reference: unrestricted sleep episodes; ^d^reference: 12h-weekend shifts (day/night)

**Table E. Linear mixed model analysis for range of LIDS oscillation stratified by chronotype**

|  | Early chronotype | | | Intermediate chronotype | | | Late chronotype | | |
| --- | --- | --- | --- | --- | --- | --- | --- | --- | --- |
| Fixed effects | β | 95% CI | | β | 95% CI | | β | 95% CI | |
| Intercept | 75.13 | 53.65 | 98.88 | 70.61 | 54.04 | 87.22 | 107.91 | 88.06 | 127.52 |
| Age (10 years) | -2.04 | -4.39 | 0.26 | 0.10 | -1.93 | 2.12 | **-5.63** | **-8.75** | **-2.47** |
| Men^a^ | -0.71 | -13.94 | 12.53 | 0.79 | -8.23 | 9.61 | -4.68 | -12.25 | 2.80 |
| Sleep duration (h) | 0.95 | -0.43 | 2.19 | 0.21 | -0.68 | 1.12 | -0.55 | -1.75 | 0.65 |
| LIDS cycle number | -7.90 | -17.43 | 0.64 | -4.43 | -10.37 | 1.52 | **-13.91** | **-22.30** | **-5.40** |
| Night sleep^b^ | 6.25 | -3.93 | 15.40 | 0.01 | -5.73 | 5.80 | -3.74 | -10.66 | 2.90 |
| Work day^c^ | ***5.18*** | ***-0.19*** | ***10.24*** | 2.49 | -1.16 | 6.17 | -2.18 | -7.31 | 2.44 |
| Roster^d^ |  |  |  |  |  |  |  |  |  |
| 12h-shifts (day/night) | -6.69 | -25.14 | 10.93 | -6.27 | -14.64 | 2.01 | -6.60 | -14.37 | 1.41 |
| 8h-shifts with night work | -11.59 | -29.64 | 5.93 | -7.53 | -15.97 | 0.84 | **-11.93** | **-19.78** | **-3.75** |
| 8h-shifts without night work | -5.03 | -22.17 | 11.60 | - | - | - | **-18.85** | **-33.86** | **-3.44** |
| Permanent night shifts | -2.47 | -24.50 | 18.67 | -2.84 | -14.42 | 8.48 | -5.77 | -12.98 | 1.78 |
| Interaction effects |  |  |  |  |  |  |  |  |  |
| Men^a^*night sleep^b^ | -1.00 | -9.91 | 8.23 | 1.13 | -4.16 | 6.41 | 5.34 | -0.47 | 11.17 |
| LIDS cycle number*age | 0.18 | -0.74 | 1.12 | -0.17 | -0.82 | 0.48 | 0.95 | -0.36 | 2.26 |
| LIDS cycle number*men^a^ | 2.60 | -2.51 | 7.62 | -0.90 | -3.81 | 2.07 | 0.58 | -2.48 | 3.67 |
| LIDS cycle number*sleep duration | 0.54 | -0.04 | 1.17 | **0.40** | **0.01** | **0.78** | 0.51 | -0.02 | 1.02 |
| LIDS cycle number*night sleep^b^ | -1.65 | -3.41 | 0.59 | -0.06 | -1.29 | 1.16 | 0.37 | -1.59 | 2.42 |
| LIDS cycle number*work day^c^ | -1.68 | -3.91 | 0.66 | -0.57 | -2.15 | 0.99 | 0.93 | -1.13 | 3.16 |
| LIDS cycle number*roster^d^ |  |  |  |  |  |  |  |  |  |
| 12h-shifts (day/night) | -2.54 | -9.52 | 4.85 | -0.75 | -3.43 | 1.95 | 1.59 | -1.83 | 4.95 |
| 8h-shifts with night work | -1.91 | -8.85 | 5.32 | 0.22 | -2.51 | 2.96 | 2.10 | -1.35 | 5.42 |
| 8h-shifts without night work | -2.77 | -9.33 | 4.06 | - | - | - | **6.68** | **0.23** | **13.00** |
| Permanent night shifts | -5.48 | -13.91 | 3.46 | -2.26 | -6.12 | 1.69 | 2.00 | -1.31 | 5.13 |

CI=95% confidence interval; ^a^reference: women; ^b^reference: no sleep between 8pm and 8am; ^c^reference: unrestricted sleep episodes; ^d^reference: 12h-weekend shifts (day/night)
